# Assessment of Problem Gambling in a Chinese Context: The Chinese G-MAP

**DOI:** 10.1100/tsw.2009.42

**Published:** 2009-07-02

**Authors:** Daniel T. L. Shek, Elda M. L. Chan

**Affiliations:** ^1^ Department of Applied Social Sciences, Hong Kong Polytechnic University, Hong Kong, China; ^2^ Department of Sociology, East China Normal University, Shanghai, China; ^3^ Kiang Wu Nursing College of Macau, Macau, China; ^4^ Even Centre, Tung Wah Group of Hospitals, Hong Kong, China

**Keywords:** problem gambling; assessment, Chinese G-MAP, addiction

## Abstract

There is a severe lack of instruments to assess problem gambling in Chinese people. This study examined the psychometric properties of the Chinese version of the Maroondah Assessment Profile for Problem Gambling (Chinese G-MAP), based on the responses of eight problem gamblers and 125 pathological gamblers seeking help from a problem gambling treatment center. Reliability analyses showed that the G-MAP and its related domains and scales were generally internally consistent. There are also several lines of evidence suggesting that the Chinese G-MAP and the various domains are valid: (a) the various G-MAP domain and scale measures were significantly correlated among themselves, (b) the G-MAP measures were significantly correlated with pathological gambling behavior assessed by the 4^th^ Edition of the Diagnostic and Statistical Manual of Mental Disorders (DSM-IV), and (c) the G-MAP total scale and domain measures were able to discriminate problem gamblers and pathological gamblers. The present study suggests that the Chinese G-MAP possesses acceptable psychometric properties that can be used in research and practice settings.

## INTRODUCTION

A survey of the literature shows that pathological gambling is a growing problem in the global context. Based on the existing prevalence studies of problem gambling in the U.S., Petry[1] reported that the prevalence rates of pathological gambling was around 1–2% of the general population, whereas subthreshold pathological gambling was around 3–4% of the general population. In Canada, Cox et al.[2] conducted a survey of the prevalence rates across 10 Canadian provinces and reported that the 12-month prevalence of gambling problems in Canada was 2.0%, with variations across different provinces. In Spain, Becona[3] reported that lifetime and past-year prevalence rates of pathological gambling were 0.92 and 0.31%, respectively. Findings on the prevalence rates of pathological gambling in Sweden and New Zealand have also been reported[4].

There are also research studies suggesting that problem gambling is a growing problem in Hong Kong. The Centre for Social Policy Studies of the Department of Applied Social Sciences and the General Education Centre of The Hong Kong Polytechnic University[5] conducted a ground-breaking study of gambling among the general public in Hong Kong. Using the criteria from the 4$^{th}$ Edition of the Diagnostic and Statistical Manual of Mental Disorders (DSM-IV) for assessing pathological gambling, the researchers found that 1.85% of the 2,004 respondents could be classified as "probable pathological gamblers" (i.e., respondents displaying five or more symptoms). Fong and Ozorio[6] conducted the first scientific study on pathological gambling in Macau. Utilizing a computer-assisted random digit dialing method (N = 1,121 interviews), results showed that 1.78 and 2.5% of the respondents could be classified as probable pathological gamblers and problem pathological gamblers, respectively.

What are the factors leading to problem gambling? With reference to the ecological models, an integration of the theories and research findings in the literature shows that several groups of factors would increase the likelihood of pathological gambling. In the first place, there are theories suggesting that cognitive biases and distortions contribute to the development of pathological gambling. For example, many pathological gamblers believe that they can beat the gambling industry and they will win despite repeated loss. Empirically, many studies have shown that experience of illusion of control and unrealistic perception of luck are prevalent in problem gamblers[7]. Second, emotional factors are related to problem gambling. There are theories suggesting that gambling is consciously or unconsciously used as a strategy to cope with negative emotions in pathological gamblers. There are also research findings showing that there are greater emotional problems (depression, suicidal ideation) in adolescent gamblers[8] and there is high impulsivity in adolescent problem gamblers[9]. Dickson et al.[10] also reported heightened risk for suicidal ideation and attempts in problem gamblers.

Third, situation factors are related to problem gambling. There are theories suggesting that inability to cope with demands in life contributes to pathological gambling. Research studies have shown that the coping behavior in adolescent gamblers is poor[8] and that problem gambling may occur after negative life events. There are also studies showing that resilience is negatively related to adolescent high-risk behavior. Fourth, there are theories suggesting that negative self-perceptions and low self-image (i.e., attitudinal factors) are intrinsic to pathological gambling. As such, pathological gamblers may make use of gambling to obtain achievement. There are research findings showing that adolescent gambling is closely related to delinquency and conduct problems, which would strengthen the negative image of pathological gamblers[11]. Lower self-esteem in adolescent problem gamblers has also been reported[10].

Finally, there are theories suggesting that interpersonal and social problems are conducive to pathological gambling. For example, many parents and friends of adolescent gamblers are gamblers. There are also research findings showing that perceived family and peer support is poor among adolescent problem gamblers[11], and that quality friendships and relationships are lost and replaced by gambling associates in pathological gamblers[12]. Finally, it was found that there is higher susceptibility to peer pressure in probable pathological gamblers[9].

Obviously, a careful assessment of the above factors in problem gamblers would be important to give a clear picture about the possible factors that are conducive to the problem, and the related assessment would give pointers to intervention and prevention at different levels. In this study, the psychometric properties of the Chinese version of the Maroondah Assessment Profile for Problem Gambling (Chinese G-MAP), based on the work of Loughnan et al.[13], would be examined. There are five domains and 17 scales in the G-MAP that resemble the five groups of factors related to pathological gambling outlined above. The domains and related scales of the G-MAP are as follows:

***Domain 1: Beliefs about Winning Domain (Cognitive Problems)***

- Control Scale: belief in the efficacy of one's system
- Prophecy Scale: use of intuition and ideas about luck to achieve successful outcomes
- Uninformed Scale: belief that gambling is a reasonable way to make money

***Domain 2: Feelings Domain (Emotional Problems)***

- Good Feelings Scale: use of gambling to lift one's mood
- Boredom Scale: use of gambling to alleviate boredom
- Numbness Scale: dissociation and disconnection from emotional responses when engaging in gambling
- Relaxation Scale: use of gambling to control stress

***Domain 3: Situations Domain (Life Situations Related to Pathological Gambling)***

- Desperation Scale: gambling as a result of financial desperation
- Transition Scale: relationship between gambling and transitional events in lives
- Oasis Scale: gambling behavior as an "escape" from the perceived demands in life (coping problem)
- Mischief Scale: gambling as a result of the dire need to be "naughty" or rebellious

***Domain 4: Attitudes to Self Domain (Self-Concept and Psychological Problem)***

- Negative Life-Script Scale: belief that others see one as a "loser" and wish that gambling can help them to be a "winner"
- Entrenchment Scale: belief that gambling is a disease or affliction that can only be solved by lifelong abstinence
- Self-Harm Scale: conscious use of gambling to punish or hurt oneself
- Winner Life-Script Scale: gambling as a result of the desire to maintain self-image of being a "winner"

***Domain 5: Social Domain (Social Influences)***

- Friendship Scale: use of gambling to increase social encounter
- Shyness Scale: use of gambling to satisfy the desire to be around people, but minimize the pressure to interact with them

Based on the responses of Chinese problem gamblers and pathological gamblers to the Chinese G-MAP, the psychometric properties of the Chinese G-MAP were examined in this study. There are two questions addressed in this study: (1) Are the Chinese G-MAP measures internally consistent? (2) Is the Chinese G-MAP valid? For the second question, the following information would be examined: (a) inter-relationships among the G-MAP measures and scales, (b) correlation between the G-MAP measures and pathological gambling behavior assessed by the DSM-IV, and (c) the ability of the G-MAP total scale and domain measures to discriminate problem gamblers and pathological gamblers.

## METHODS

### Participants and Procedures

The present study was based on the clients seeking help from the Even Centre, Tung Wah Group of Hospitals (N = 134). When these clients sought help from the Even Centre, they were requested to complete a questionnaire with the following sections:

1. Demographic information (e.g., gender, age, and marital status)
2. Presenting problems (e.g., gambling problem, occupational problem, suicidal behavior, and interpersonal problems)
3. Problems arising from debts
4. Whether family members and friends are helping the client to pay off his/her debts
5. Employment status

6. Source of income
7. Monthly income
8. Age of first gambling
9. Gambling age (i.e., duration of gambling)
10. Duration of problem gambling
11. Gambling activities leading to gambling problems
12. Current gambling situation
13. Gambling history
14. Family background and structure
15. Personal background and developmental history
16. Health and mental health conditions
17. Whether the client is receiving psychiatric treatment
18. Risk assessment
19. Problem behavior (e.g., substance abuse, the Net addiction, criminal history)
20. Self-assessment of gambling problem
21. Assessment based on the G-MAP
22. Proposed treatment plan

Besides the above self-report questionnaire, the participants were also assessed by the workers using the diagnostic criteria for pathological gambling based on the DSM-IV. If a participant endorsed three to four criteria, he/she was considered to have problem gambling. If a participant endorsed five or more criteria, the person was regarded to have pathological gambling.

The client was also requested to complete the Chinese G-MAP. The G-MAP is an 85-item self-report inventory that assesses 17 aspects associated with problem gambling. The derivation of the 17 aspects of the tool was based on the clinical experiences and related discussion of the three clinicians who developed the tool[13]. According to the test developers, the related 17 scales could be further grouped into five domains.

The psychometric properties of the Chinese G-MAP were examined in this study. For the reliability of the tool, the internal consistency of the G-MAP total scale, five domain scales, and the 17 individual scales were examined. For the validity of the G-MAP measures, it was expected that different G-MAP domains and scales would have significant correlations among themselves. As the G-MAP measures (e.g., biased beliefs about gambling) are theoretically related to gambling behavior assessed by the diagnostic criteria of the DSM-IV, it would be expected that the G-MAP measures would be significantly related to the DSM-IV assessment scores (i.e., construct validity). Finally, based on the classification of gamblers using the DSM-IV, it would be expected that different types of gamblers would have different scores on the G-MAP measures (i.e., criterion-related validity).

## RESULTS

Based on the operational definition that those who endorsed five or more symptoms were regarded as “pathological gamblers”, 94.0% of the respondents (N = 125) could be regarded as pathological gamblers and 6% of them (N = 8) could be regarded as problem gamblers.

Concerning the reliability of the Chinese G-MAP, the findings showed that the G-MAP measures were basically internally consistent (Table 1). With reference to the 85-item Chinese G-MAP, results showed that the scale was internally consistent (alpha = 0.96 and mean interitem correlation = 0.21). With the exception of items 4, 7, 38, and 55, the values of the item-total correlation coefficients were acceptable. For the different G-MAP domains, the domain scales were internally consistent.

**TABLE 1**
**Coefficient Alpha and Mean Item-Item Correlation Coefficient for the G-MAP Domains and Scales**

| Measures | Coefficient Alpha |
|---|---|
| Beliefs about Winning Domain | 0.62 |
| Control Scale | 0.87 |
| Prophecy Scale | 0.69 |
| Uninformed Scale | 0.82 |
| Feelings Domain | 0.82 |
| Good Feelings Scale | 0.80 |
| Relaxation Scale | 0.75 |
| Boredom Scale | 0.83 |
| Numbness Scale | 0.57 |
| Situations Domain | 0.82 |
| Oasis Scale | 0.73 |
| Transition Scale | 0.83 |
| Desperation Scale | 0.73 |
| Mischief Scale | 0.73 |
| Attitudes to Self Domain | 0.68 |
| Negative Life-Script Scale | 0.78 |
| Winner Life-Script Scale | 0.70 |
| Entrenchment Scale | 0.42 |
| Self-Harm Scale | 0.66 |
| Social Domain | 0.83 |
| Friendship Scale | 0.68 |
| Shyness Scale | 0.72 |
| G-MAP | 0.96 |

For the validity of the G-MAP, there are several lines of evidence suggesting that the tool and the related measures are reliable. First, assuming that the scales in each domain assess similar traits, it would be expected that the different scales and domains would be inter-related. The findings are basically consistent with this general expectation. The relevant findings are presented in Table 2. Second, based on the predictions of different models, it would be expected that there would be inter-relationships among the G-MAP domains. The related findings are presented in Table 3. Third, using pathological gambling defined by the DSM-IV[14] as the criterion measure, findings showed that the G-MAP scores were generally correlated with the DSM-IV scores (Table 4 and Table 5). Finally, the findings in Table 6 show that, with the exception of some individual scales, the scale, domain, and total scale measures of the Chinese G-MAP were able to discriminate pathological gamblers and problem gamblers.

## DISCUSSION

As far as the reliability of the G-MAP measures is concerned, the internal consistency of the G-MAP total scale, five domain scales, and the 17 individual scales was generally established in the present study. With the exception of the Numbness Scale and Entrenchment Scale, the interitem correlation, item-total correlation, and coefficient alpha statistics related to each measure were acceptable[15].

**TABLE 2**
**Inter-Relationships of the G-MAP Scales in Each Domain**

| | | | | | | | |
|---|---|---|---|---|---|---|---|
| Domain 1 | Control | | Prophecy | | Uniformed | | |
| Control | 1 | | | | | | |
| Prophecy | 0.29 | ** | 1 | | | | |
| Uniformed | 0.39 | *** | 0.42 | *** | 1 | | |
| Domain 2 | Good feelings | | Boredom | | Numbness | | Relaxation |
| Good Feelings | 1 | | | | | | |
| Boredom | 0.59 | *** | 1 | | | | |
| Numbness | 0.43 | *** | 0.33 | *** | 1 | | |
| Relaxation | 0.77 | *** | 0.57 | *** | 0.47 | *** | 1 |
| Domain 3 | Desperation | | Transition | | Oasis | | Mischief |
| Desperation | 1 | | | | | | |
| Transition | 0.51 | *** | 1 | | | | |
| Oasis | 0.49 | *** | 0.61 | *** | 1 | | |
| Mischief | 0.40 | *** | 0.54 | *** | 0.71 | *** | 1 |
| Domain 4 | Negative Life-Script | | Entrenchment | | Self-Harm | | Winner Life-Script |
| Negative Life-Script | 1 | | | | | | |
| Entrenchment | 0.21 | * | 1 | | | | |
| Self-Harm | 0.35 | *** | 0.07 | ns | 1 | | |
| Winner Life-Script | 0.70 | *** | 0.26 | ** | 0.35 | *** | 1 |
| Domain 5 | Friendship | | Shyness | | | | |
| Friendship | 1 | | | | | | |
| Shyness | 0.72 | *** | 1 | | | | |

*$p < 0.05$; **$p < 0.01$; ***$p < 0.001$; ns, nonsignificant.

**TABLE 3**
**Correlations Among the G-MAP Domain Measures**

| | Beliefs about Winning Domain | | Feelings Domain | | Situations Domain | | Attitudes to Self Domain | | Social Domain | | G-MAP |
|---|---|---|---|---|---|---|---|---|---|---|---|
| Beliefs about Winning Domain | 1 | | | | | | | | | | |
| Feelings Domain | 0.39 | *** | 1 | | | | | | | | |
| Situations Domain | 0.54 | *** | 0.67 | *** | 1 | | | | | | |
| Attitudes to Self Domain | 0.65 | *** | 0.66 | *** | 0.77 | *** | 1 | | | | |
| Social Domain | 0.48 | *** | 0.68 | *** | 0.68 | *** | 0.69 | *** | 1 | | |
| G-MAP | 0.71 | *** | 0.84 | *** | 0.90 | *** | 0.90 | *** | 0.82 | *** | 1 |

*$p < 0.05$; **$p < 0.01$; ***$p < 0.001$; ns, nonsignificant.

**TABLE 4**
**Correlation Coefficients on the Relationships Between the Chinese G-MAP Measures and the DSM-IV Measures**

| Measure | DSM-IV Total Score | | DSM-IV Classification Score | |
|---|---|---|---|---|
| Control | 0.10 | ns | 0.14 | ns |
| Prophecy | 0.08 | ns | 0.12 | ns |
| Uninformed | 0.31 | *** | 0.33 | *** |
| Good Feelings | 0.26 | ** | 0.16 | ns |
| Boredom | 0.24 | ** | 0.17 | * |
| Numbness | 0.10 | ns | –0.04 | ns |
| Relaxation | 0.26 | ** | 0.21 | * |
| Desperation | 0.46 | *** | 0.26 | ** |
| Transition | 0.26 | ** | 0.18 | * |
| Oasis | 0.31 | *** | 0.18 | * |
| Mischief | 0.27 | ** | 0.08 | ns |
| Negative Life-Script | 0.17 | * | 0.17 | * |
| Entrenchment | 0.20 | * | 0.11 | ns |
| Self-Harm | 0.06 | ns | –0.10 | ns |
| Winner Life-Script | 0.17 | * | 0.19 | * |
| Friendship | 0.27 | ** | 0.23 | ** |
| Shyness | 0.28 | ** | 0.19 | * |
| G-MAP | 0.34 | *** | 0.24 | *** |

*$p < 0.05$; **$p < 0.01$; ***$p < 0.001$; ns, nonsignificant.

**TABLE 5**
**Correlation Coefficients on the Relationships Between the Chinese G-MAP Domain Measures and the DSM-IV Measures**

| Measure | DSM-IV Total Score | | DSM-IV Classification Score | |
|---|---|---|---|---|
| Beliefs about Winning Domain | 0.21 | * | 0.26 | ** |
| Feelings Domain | 0.27 | ** | 0.16 | ns |
| Situations Domain | 0.40 | *** | 0.22 | * |
| Attitudes to Self Domain | 0.21 | * | 0.15 | ns |
| Social Domain | 0.29 | ** | 0.23 | ** |

*$p < 0.05$; **$p < 0.01$; ***$p < 0.001$; ns, nonsignificant.

In line with our expectation, the G-MAP domains and scales were significantly correlated among themselves. This observation suggests that the cognitive, emotional, attitudinal, and interpersonal factors are inter-related. In the long run, it would be theoretically interesting to examine the inter-relationships among the different domains. For the microtheories, it would be expected that inner problems (e.g., cognitive problems) would be precursors of situation problems (e.g., desperation). On the other hand, extraindividual theories would predict that environmental problems (e.g., family issues) would lead to inner problems.

**TABLE 6**
**Differences Between Problem Gamblers and Pathological Gamblers on the G-MAP Measures**

| Measures | Problem Gamblers | | Pathological Gamblers | | t Value | |
|---|---|---|---|---|---|---|
| | Mean | S.D. | Mean | S.D. | | |
| Control Scale | 7.13 | 5.84 | 10.52 | 5.64 | –1.65 | * |
| Prophecy Scale | 8.38 | 4.03 | 10.51 | 4.31 | –1.37 | ns |
| Uniformed Scale | 8.25 | 4.13 | 14.54 | 4.37 | –3.96 | *** |
| Good Feelings | 6.38 | 4.98 | 9.83 | 5.01 | –1.89 | * |
| Boredom | 6.88 | 4.97 | 10.76 | 5.32 | –2.01 | * |
| Numbness | 9.00 | 4.28 | 8.28 | 4.27 | 0.46 | ns |
| Relaxation | 5.75 | 4.13 | 10.02 | 4.88 | –2.41 | ** |
| Desperation | 3.25 | 3.49 | 8.80 | 4.91 | –3.14 | ** |
| Transition Scale | 4.25 | 6.07 | 8.37 | 5.50 | –2.04 | * |
| Oasis Scale | 3.88 | 4.09 | 7.35 | 4.65 | –2.06 | * |
| Mischief Scale | 4.50 | 4.41 | 6.03 | 4.60 | –0.92 | ns |
| Negative Life-script Scale | 5.75 | 3.85 | 9.66 | 5.36 | –2.02 | * |
| Entrenchment | 11.25 | 4.50 | 12.94 | 3.44 | –1.32 | ns |
| Self-Harm | 5.63 | 5.80 | 4.02 | 3.81 | 1.11 | ns |
| Winner Life-Script Scale | 5.50 | 4.00 | 9.32 | 4.75 | –2.22 | * |
| Friendship Scale | 2.00 | 1.85 | 5.98 | 4.15 | –2.69 | ** |
| Shyness Scale | 4.88 | 4.82 | 8.43 | 4.39 | –2.21 | * |
| Belief about Winning Domain | 23.75 | 6.84 | 35.58 | 10.94 | –3.01 | ** |
| Feelings Domain | 28.00 | 16.13 | 38.89 | 15.67 | –1.90 | * |
| Situations Domain | 15.88 | 13.64 | 30.55 | 15.92 | –2.55 | ** |
| Attitudes to Self Domain | 28.13 | 9.52 | 35.94 | 12.76 | –1.70 | * |
| Social Domain | 6.88 | 5.79 | 14.42 | 7.92 | –2.65 | ** |
| G-MAP | 102.63 | 43.54 | 155.77 | 52.73 | –2.79 | ** |

*$p < 0.05$ (one-tailed); **$p < 0.01$ (one-tailed); ***$p < 0.001$ (one-tailed); ns, nonsignificant.

Consistent with the expectation that the G-MAP measures would be significantly related to the DSM-IV scores, the findings showed that there was a general relationship between these two domains, thus providing support for the construct validity of the G-MAP measures. Finally, the findings showed that different types of gamblers had different scores on the G-MAP measures, thus providing support for the criterion-related validity of the measures. In short, there is support for the different aspects of the validity of the G-MAP. Given the small sample size in the total sample and the problem gambler sample, the present findings can be regarded as robust and encouraging. In view of the paucity of psychosocial assessment tools in the Chinese context for problem gambling, this study is an important addition to the literature[16,17]. The present work is also important in view of the growing addiction culture in Hong Kong[18] and there is a call for evidence-based welfare services in Hong Kong[19].

Although the present study provides some support for the reliability and validity of the Chinese G-MAP measures, further information should be collected for the test-retest reliability of the measures. Furthermore, additional studies on the convergent and discriminant validities of the Chinese G-MAP measures should be conducted in future. Finally, data collected from community samples would also substantiate the research and clinical utility of the G-MAP in the Chinese culture.
